# The Influence of a Memory Delay on Spatial Coding in the Superior Colliculus: Is Visual Always Visual and Motor Always Motor?

**DOI:** 10.3389/fncir.2018.00074

**Published:** 2018-10-22

**Authors:** Morteza Sadeh, Amirsaman Sajad, Hongying Wang, Xiaogang Yan, John Douglas Crawford

**Affiliations:** ^1^York Centre for Vision Research, York University, Toronto, ON, Canada; ^2^Vision: Science to Applications (VISTA) Program, York University, Toronto, ON, Canada; ^3^York Neuroscience Graduate Diploma Program, York University, Toronto, ON, Canada; ^4^Canadian Action and Perception Network (CAPnet), York University, Toronto, ON, Canada; ^5^Departments of Psychology, Biology and Kinesiology and Health Science, York University, Toronto, ON, Canada

**Keywords:** superior colliculi, saccades, primates, memory, gaze shift

## Abstract

The memory-delay saccade task is often used to separate visual and motor responses in oculomotor structures such as the superior colliculus (SC), with the assumption that these same responses would sum with a short delay during immediate “reactive” saccades to visual stimuli. However, it is also possible that additional signals (suppression, delay) alter visual and/or motor response in the memory delay task. Here, we compared the spatiotemporal properties of visual and motor responses of the *same* SC neurons recorded during both the reactive and memory-delay tasks in two head-unrestrained monkeys. Comparing tasks, visual (aligned with target onset) and motor (aligned on saccade onset) responses were highly correlated across neurons, but the peak response of visual neurons and peak motor responses (of both visuomotor (VM) and motor neurons) were significantly higher in the reactive task. Receptive field organization was generally similar in both tasks. Spatial coding (along a Target-Gaze (TG) continuum) was also similar, with the exception that pure motor cells showed a stronger tendency to code future gaze location in the memory delay task, suggesting a more complete transformation. These results suggest that the introduction of a trained memory delay alters both the vigor and spatial coding of SC visual and motor responses, likely due to a combination of saccade suppression signals and greater signal noise accumulation during the delay in the memory delay task.

## Introduction

The primate superior colliculus (SC) has been studied extensively both for its specific role in generating saccades and head-unrestrained gaze shifts, and as a general model for sensory-motor transformations (Mays and Sparks, 1980; Wurtz and Albano, 1980; Optican, 1995; Marino et al., 2008; Sadeh et al., 2015). One defining characteristic of the SC is that its neurons can be categorized into populations with only “visual” responses (briefly delayed burst responses to a visual stimulus), only “motor” responses (burst activity just before and after a saccade) or visuomotor (VM) responses, i.e., both visual and motor (Wurtz and Goldberg, 1972a,b; Sparks, 1978; Harris, 1980; Wurtz and Albano, 1980; Bruce and Goldberg, 1985; Bruce et al., 1985; Munoz and Wurtz, 1995a,b; Stricanne et al., 1996; Freedman and Sparks, 1997a; Gandhi and Katnani, 2011; Bremmer et al., 2016). Implicit in this categorization is the assumption that these responses are task-independent, but this is not necessarily the case. Here, we specifically examined whether the task typically used to separate these cell types might itself influence their neural code, and conversely, whether these responses code something different in simpler gaze saccades.

The typical way to separate visual and motor responses is to introduce a memory delay between a transient visual stimulus and the gaze saccade (Wurtz and Goldberg, 1972a; Sparks, 1978; Wurtz and Albano, 1980; Sparks and Hartwich-Young, 1989; Stanford and Sparks, 1994; Munoz and Wurtz, 1995a,b; Sajad et al., 2015). Delays of 500–1500 ms provide a clean temporal segregation between the visual response and/or motor response. The addition of a spatial separation between the visual stimulus and the saccade vector across the memory delay (e.g., using a double step saccade task or the anti-saccades task) likewise separate the spatial tuning of visual and motor responses (Everling and Munoz, 2000; Munoz and Everling, 2004) More recently, we have shown that even in the absence of these spatial manipulations, the SC visual response encodes target location relative to initial eye orientation whereas after a memory delay the motor response encodes future gaze direction relative to current eye orientation (Sadeh et al., 2015).

Saccades made immediately and directly to a transient visual stimulus, without a memory delay, are called “reactive saccades” (Sparks, 1978; Pierrot-Deseilligny et al., 1991a; Deubel, 1995; Munoz and Wurtz, 1995a,b). During such saccades there is typically a 25–50 ms peak-to-peak delay between visual and motor responses, although this is prolonged during head unrestrained gaze shifts (Freedman, 2008; DeSouza et al., 2011; Sadeh et al., 2018). In either case, there is significant temporal overlap between these responses. As a result, VM cells often show a continuous burst, but often with a slight inflection between peaks that correspond in time to the visual and motor burst (Sparks, 1978; Munoz and Wurtz, 1995a,b; Dorris et al., 1997; DeSouza et al., 2011). Often, investigators use such inflections to arbitrarily draw a “line” between visual and motor responses, knowing very well that they might actually blend into each other (Mays and Sparks, 1980; Everling et al., 1999; Marino et al., 2008, 2015; DeSouza et al., 2011; Sadeh et al., 2015). It is generally assumed that these responses summate linearly in reactive saccades. This assumption seems to be supported by our recent finding that during reactive saccades, SC cells show a transition from target coding in their visual response to gaze coding in their motor responses (Sadeh et al., 2018) similar to that observed previously after a memory delay (Sadeh et al., 2015). However, in the absence of a direct comparison, it cannot be assumed that the spatial codes of SC visual and motor responses are quantitatively identical both with and without a memory delay.

First, the temporal overlap between visual and motor signals in VM cells might influence their respective codes. For example, VM cells showed a progressive transition between intermediate target-gaze codes during both reactive and memory delay saccades, but with very different time courses (Sadeh et al., 2015, 2018). This could lead to greater overlap in VM cell codes in the reactive task. Conversely, the addition of a memory delay likely introduces additional signals that could influence spatial codes. These include saccade suppression signals that could influence the vigor of both visual and motor responses (Thiele et al., 2002; Munoz and Everling, 2004), and memory delay/motor build up activity that might specifically influence the final motor response (Munoz and Wurtz, 1995a,b; Miller et al., 1996; Pesaran et al., 2002; Sajad et al., 2016a,b). In the past it was not possible to test all of these predictions, because the technology was lacking to probe specific VM codes in the absence of additional spatial manipulations.

In the current study, we investigated if the visual and motor responses observed in reactive saccades altered, either in amplitude or spatial content, by the insertion of a memory delay. To do this, we recorded from the same SC neurons using both the reactive and memory delay tasks, and analyzed and directly compared their firing rates and spatial content. We did this using an analytic approach based on variable gaze errors that allowed us to fit activity from specific visual and motor response epochs against models along a visual-motor continuum (Keith and Crawford, 2008; DeSouza et al., 2011; Sadeh et al., 2015; Sajad et al., 2015). This was done in head unrestrained animals because this reflects a more natural behavioral condition, allowed us to eliminate some other models in our initial analysis (Sadeh et al., 2015), and in this specific case provided more prolonged and temporally rich response profiles for our analysis. We found that, although certain fundamental aspects are retained in visual and motor responses (such as the preference for target vs. gaze coding) the addition of a memory delay does introduce subtle alterations to the amplitudes and spatial codes of SC signals, particularly in the motor responses, which may influence behavior.

## Materials and Methods

### Animals and Surgical Procedures

The data were collected from two female monkeys (Macaca Mulatta, M1 and M2; age, 10 years; weights, 6.5 and 7 kg) with a protocol approved by the York University Animal Care Committee in accordance with guidelines published by the Canadian Council for Animal Care. With similar surgical procedures as described previously (Crawford et al., 1999; Klier et al., 2001), the monkeys were prepared for long-term electrophysiology and 3D gaze movement recordings. Each monkey was subjected to general anesthesia with 1%–2% isoflurane after intramuscular injection of ketamine hydrochloride (10 mg/kg), atropine sulfate (0.05 mg/kg) and acepromazine (0.5 mg/kg). To minimize the collisions between experimental setup and Microdrive/electrode we implanted a vertically aligned unit recording chamber (i.e., with no tilt) placed 5 mm anterior and 0 mm lateral in stereotaxic coordinates, which allowed access to the left and right SC. This chamber angle and position were chosen to minimize collisions between the electrode/microdrive and the experimental setup during head movements, and to simplify the use of stereotaxic coordinates during recordings. The chamber was then surrounded by a dental acrylic cap, which was anchored to the skull with 13 stainless steel cortex screws. Two scleral search coils (diameter, 5 mm) were implanted in one eye of the monkeys to record 3D eye movements. Two orthogonal coils, which were secured with a screw on a plastic base on the cap, recorded the 3D head movements during the experiments. 3D recordings and analysis were performed as described previously (Crawford et al., 1999; DeSouza et al., 2011).

### Experimental Equipment

We used a Pentium IV PC and custom-designed software to present stimuli, control behavior paradigms, send digital codes to a Plexon data acquisition system, and deliver juice rewards to the monkeys. Stimuli were presented on a screen 60 cm in front of the monkey, by use of a projector (WT600 DLP projector; NEC). Monkeys were seated on a custom-designed primate chair in order to have their heads move freely at the center of a 1-m^3^ magnetic field generator (Crawford et al., 1999) and a juice spout (Crist Instruments) was placed on the skull cap for computer-controlled delivery of the juice reward to the monkey’s mouth.

### Behavioral Recordings ad Paradigms

All experiments were performed using 3D recordings in head-unrestrained conditions (Crawford et al., 1999; Sadeh et al., 2015). Head motion was not analyzed in the current experiment, but provided some advantages: for comfort, natural system behavior, adequate range of gaze motion for testing large neural response fields (RF; see below). Conversely, 3D recordings and analysis were required, to account for the significant torsional eye rotation and prominent non-linearities that occur in the head unrestrained gaze range (Tweed and Vilis, 1990; Crawford et al., 1999; Klier et al., 2001; Keith et al., 2009; DeSouza et al., 2011). The target-relative-to-eye (Te) and gaze-relative-to-eye (Ge) models tested in this study were computed by rotating (not subtracting) a vector pointing from the eye toward the target or future gaze position by the inverse of initial 3D eye orientation (Klier et al., 2002).

All neurons described in the current study were tested in both of the following two paradigms.

#### Reactive Task (Figures 1A,C)

**Figure 1:**
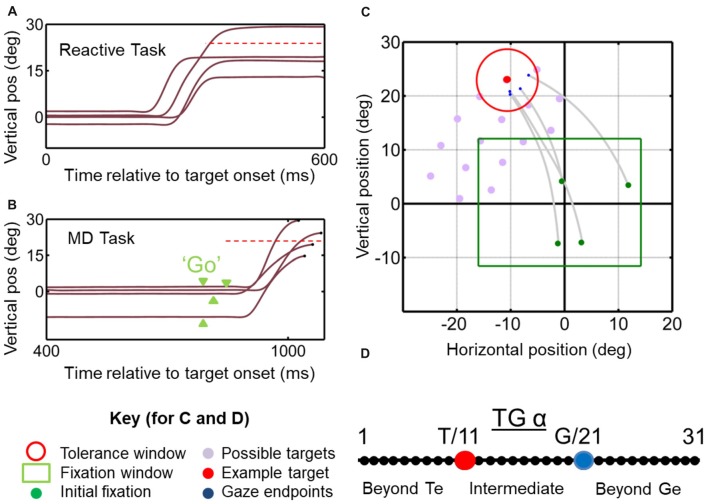
Temporal **(A,B)** and spatial **(C,D)** aspects of the behavioral tasks. **(A)** Vertical gaze position toward an upward target (dashed red horizontal line) plotted as a function of time for example trials in the *reactive task*. Results from this task are reported in Sadeh et al. (2018). **(B)** Similar gaze traces for the same target, but obtained from the *memory delay task*. Note that the memory delay is variable, so the “go” signal (extinction of the fixation point) occurred at different time points (green arrow heads). Results from this task were reported in detail previously (Sadeh et al., 2015). **(C)** Two-dimensional gaze trajectories (gray lines) from the *reactive task* for an example target in monkey M2. Also shown are the range of initial fixation positions (green square), the tolerance window (red circle) and the other possible targets used in this experimental session (gray circles) to map a neuron’s receptive field. The identical spatial layouts were used for both tasks to test each neuron. **(D)** Target-Gaze (TG) continuum constructed between and beyond target position (red dot) and gaze end point (blue dot) for each trial, and used to determine best fits for neural receptive fields.

Animals were trained to fixate a central range of positions for 900–1000 ms (randomly varied interval). A tolerance window of 2–4° (radius) with respect to the fixation position was required during this period. Simultaneous with initial fixation point disappearance-serving as GO signal—a target (red circle with a size of 0.5°) was presented in the periphery for 125 ms, at locations selected for RF mapping (Figure 1C; see below for details). Note that the reason is that we aimed to control initial gaze to separate gaze-centered vs. space-centered responses, therefore we allowed the animal to produce variable final gaze errors in order to separate the T and G models as described in the current analysis. The initial fixation range is not a tolerance window; it is basically a range of an area which possible initial fixation positions (i.e., green circles) may appear in a random fashion. Animals were then required to make a gaze shift toward the briefly flashing stimulus and fixate on it for 200 ms in order to receive juice reward. In order to spatially separate targets vs. gaze coding, we designated a relatively wide tolerance window of 6–12° (diameter) for gaze errors around the locations of the targets, and thus allowed monkeys to produce a self-selected distribution of gaze end point errors around the targets. Also, every trial was inspected, and any trial in which the gaze shift was anticipated (reaction time of <100 ms after the go signal) was excluded from the analysis (see Figures 1A,C,D).

A total of 13,068 trials were completed in each of the tasks, of these 1,555 trials (11.9%) were excluded -based on the exclusion criteria explained above-in the reactive task and 1,921 (14.7) were excluded in the MD task.

#### Memory Delay Task (Figure 1B)

The conditions, fixation point and stimulus characteristics in this task were identical to the reactive task except that after 300 ms of fixation, a target stimulus appeared in the periphery for 125 ms. The fixation light remained on for another 400–700 ms in order to introduce a variable memory delay and discourage anticipation of the go signal. In addition, every trial was inspected, and any trial in which the gaze shift was anticipated (reaction time of <100 ms after the go signal) was excluded from the analysis. When the GO signal was presented, the monkeys made a gaze shift towards the remembered location of the target, and were required to maintain fixation for at least 200 ms at that final position to obtain the juice reward.

Data from these two tasks were described previously (Sadeh et al., 2015, Sadeh et al., submitted), but this is the first time that we provide a direct quantitative comparison.

### Off-Line Trial Definition and Inclusion Criteria

During our off-line analysis the beginning of a trial was defined by the appearance of the initial fixation point. The beginning of the gaze saccade was defined as the instant when its velocity exceeded 50°/s, and its end when its velocity decreased to 30°/s. All trials were considered for analysis irrespective of whether the monkey received a reward after the trial. We excluded trials based on spatial and temporal criteria. First, trials in which the directions of the gaze shifts were completely unrelated to the direction of the target (e.g., opposite direction) were removed. Then, we obtained the regression between errors in gaze vs. retinal error (the retinal angle between the fovea and the target at the initial position before the gaze shift), and removed trials with gaze error two standard deviations greater than this regression line. Furthermore, every trial was visually inspected, and any trial in which the gaze shift was anticipated (reaction time of <100 ms after the go signal) and when the gaze shift consisted of multistep saccades was excluded from the analyses described below.

### Neural Recordings and Receptive Field Mapping

We recorded extracellular activity from the left and right SC with tungsten microelectrodes (FHC). The electrode was inserted through a guide tube, which was controlled by a hydraulic microdrive (MO-90S; Narishige International, East Meadow, NY, USA). Isolated signals were amplified, filtered and stored for off-line sorting with the Plexon MAP system. The SC was identified according to criteria published previously (DeSouza et al., 2011; Sadeh et al., 2015). The steps of SC identification and confirmation are identical to those explained previously (Sadeh et al., 2015). Once an SC neuron was isolated, the target stimuli were presented in the visual field contralateral to the hemi field of the recording site to begin RF mapping. RFs were estimated through initial mapping, which involved monkeys performing visually guided saccades to a wide range of stimuli presented on the screen while cell activity was monitored on-line. Test stimuli were then selected within a grid (12–32 targets, depending on the RF size) that extended just beyond the cell’s receptive field. Figure 1C illustrates spatial the array of target used for one particular cell in the reactive task. During testing, stimuli were presented in a randomized order, and each target was presented for at least seven gaze shifts. The *MD* task was done first in all experiment sessions in order to separate the visual and motor bursts and characterize the neuron type, the behavioral task that ran afterwards were randomized for each given experiment session.

### Neuron Classification

The memory delay saccade task was used to dissociate between visual and movement related activities and categorize cells into visual, visuomotor (VM) and motor neurons. Visual neurons were defined as cells that showed a robust burst of activity (>50 spikes/s above the baseline) 40–60 ms after the stimulus presentation that lasted for ~180 ms afterwards (Goldberg and Wurtz, 1972). Motor neurons were those with robust activity or a buildup of activity peaking around the time of gaze onset, with activity starting prior to the gaze onset (100–40 ms before saccade), and that continued to ~100 ms after gaze onset. Neurons that met both of these criteria were classified as VM. When we refer to “number of spikes” below, this refers to number of action potentials in these defined temporal windows, also we use neural activity and burst interchangeably to refer to the same concept of action potentials.

### Temporal Windows for Neural Analysis

**Figure 2:**
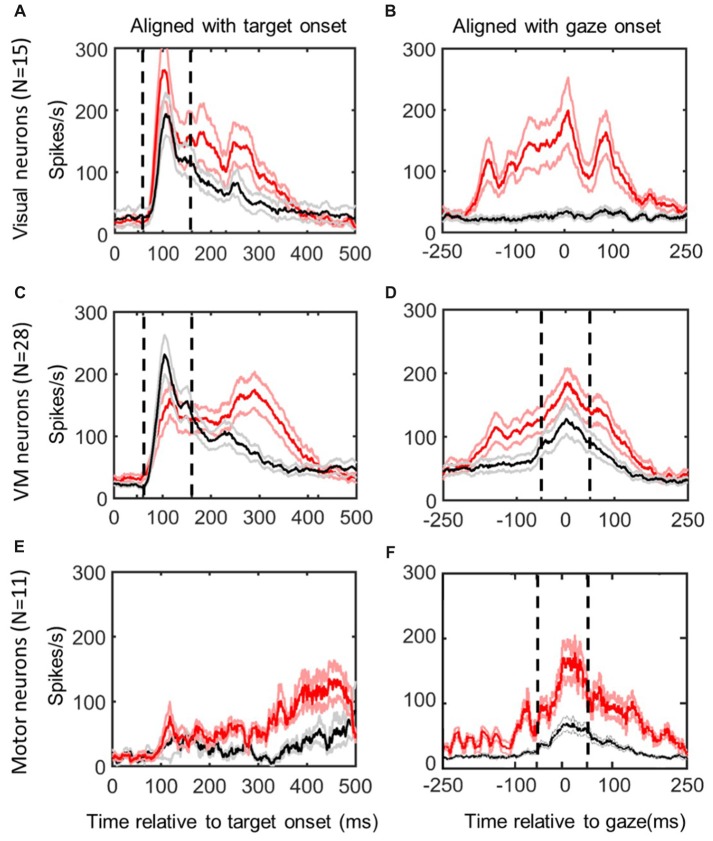
Mean spike density plots/10% confidence intervals from the same neurons in *reactive task* (red/pink) vs. the memory delay task (black/gray). Data aligned with stimulus onset (left column) and gaze movement (right column). *Top row* **(A,B)**: visual neurons, *N* = 15; *middle row* **(C,D)**: visuomotor (VM) neurons (*N* = 28); bottom row **(E,F)**: motor neurons (*N* = 11), identified using the memory delay task (Sadeh et al., 2015). Dashed vertical lines indicate the time intervals used for the “fixed window” visual (60–160 ms relative to target presentation) and motor (−50 to +50 ms relative to gaze onset) analyses.

**Figure 3:**
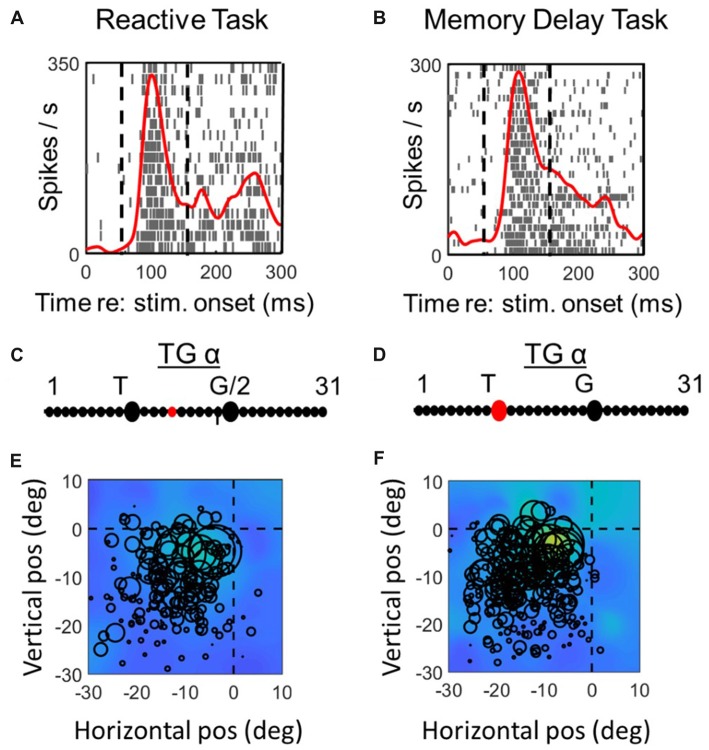
Spatial analysis of *visual* activity during *reactive task* (left column) vs. *memory delay task* for one example *visual* neuron. *Top row* **(A,C)**: spike density and raster plot aligned with target onset. Vertical dashed lines represent the fixed window of activity which was considered in our visual analysis (60–160 ms relative to target presentation). *Second row* **(B,D)**: point of best fit (red dot) on the TG continuum for the response. *Bottom row* **(E,F)**: response fields (RFs) plotted according to the best TG fit from middle row, note that the circles represent the total number of spikes in the time window for each trial (with larger circles indicating more spikes) and the overall similarity of the circle sizes for a given point in space indicate the coherency and the quality of fit (See “Materials and Methods” section). The heat maps in the background represent the non-parametric RF fits made to these data. Thus, lighter colors/larger circles indicate the “hot spots” of the receptive field.

The temporal windows that we used for analysis of bursting activity are illustrated in the results section (Figure 2). Although we observed a variable range of burst onset for visual activities which reflects different subtypes of visual and VM neuron with inputs from different brain areas, we used a fixed window of +60 to +160 ms relative to visual target presentation for visual activity based on both the visual inspection of spike density plots as well as an objective approach explained here. A time window of ±50 ms relative to saccade onset was considered for the motor activity. The fixed time windows are marked by black dashed vertical lines on Figures 3, 5, 6.

**Figure 4:**
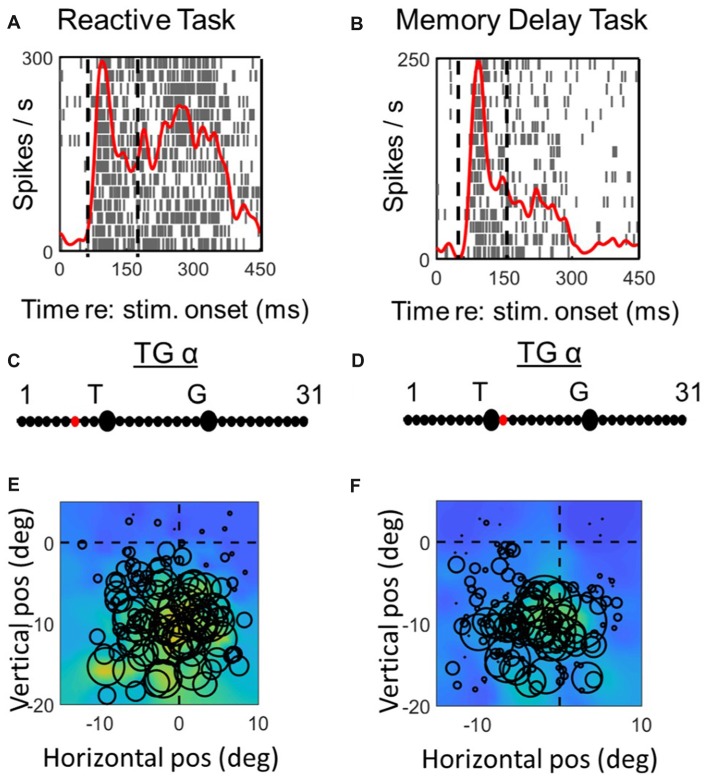
Spatial analysis of *visual* activity during *reactive task* (left column) vs. *memory delay task* for one example *VM* neuron. The plotting conventions are the same as in Figure 3.

**Figure 5:**
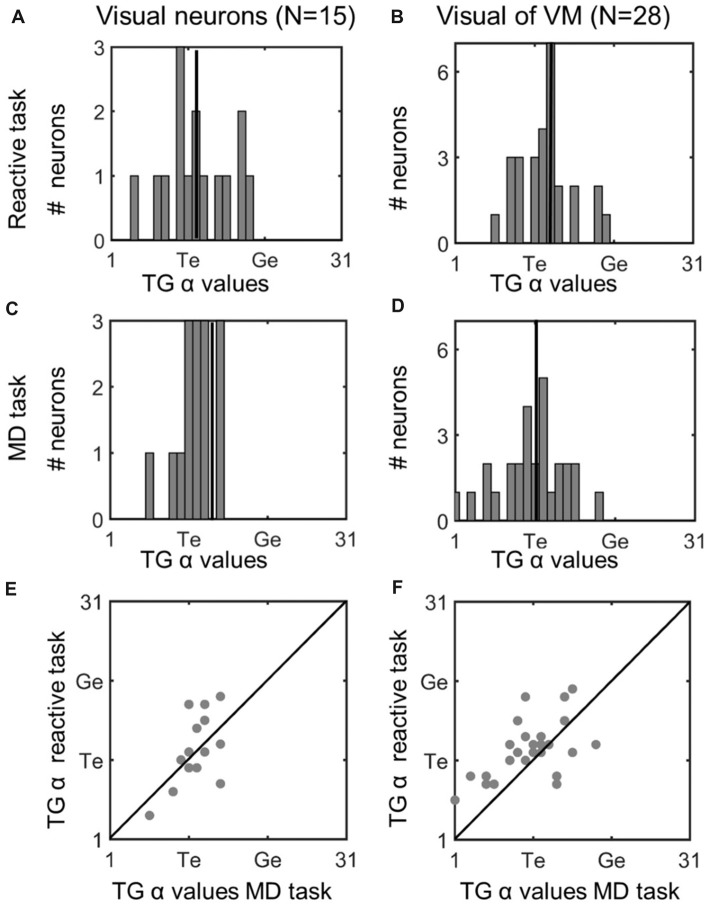
Comparison of TG continuum coding of reactive vs. memory delay task in visual responses. *Left column* **(A,C,E)**: visual neurons; *right column* **(B,D,F)**: visual response of VM neurons. Top row **(A,B)**: TG value distributions in the *reactive task.* Vertical line indicates the median. Second row **(C,D)**: distributions for same neurons in the memory delay (MD) task. Visual neurons showed a more restricted distribution, but there was no significant difference between the TG values between the two tasks. Bottom row **(E,F)**: illustrates neuron-by-neuron comparison between the two tasks in the visual activity of Visual and VM neurons, respectively, plotting the TG continuum values from the reactive task as a function of the MD task.

**Figure 6:**
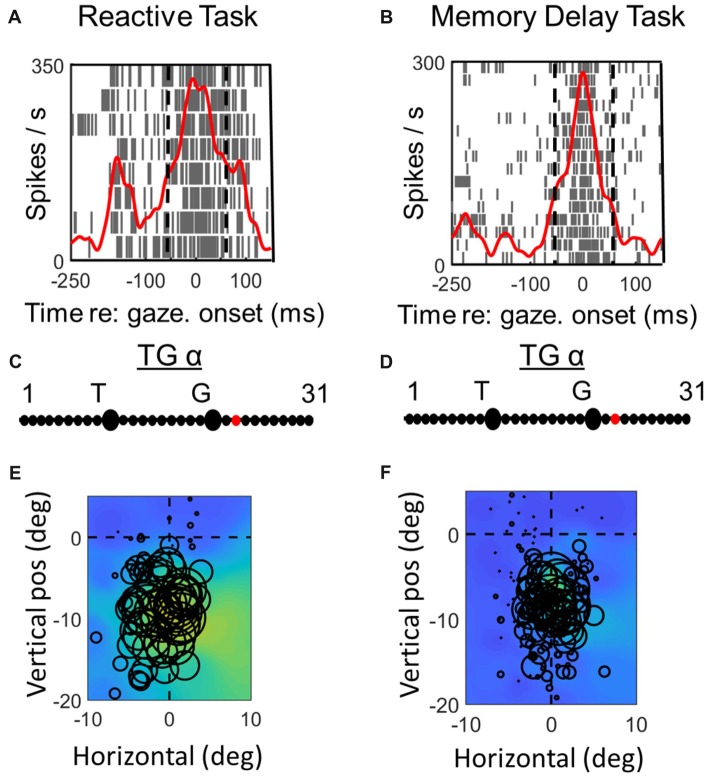
Spatial analysis of *motor* activity during reactive task (left column) vs. memory delay task for one example *VM* neuron. Vertical dashed lines in **(A,B)** represent the fixed motor analysis windows (−50 to + 50 ms relative to gaze onset). Otherwise the plotting conventions are the same as in Figure 3.

To use an objective method of determining and comparing the onset of visual bursts in each task, we used a method developed and reported by Legéndy and Salcman (1985) and applied to neural recording data for identifying burst onset by Hanes et al. (1995) as well as Thompson et al. (1996). Briefly this approach assumes that spike activity behaves in a Poisson manner (which is now generally accepted) and for every trial calculates the periods of spiking activity in which the spikes are too close in time, surpassing that described by chance levels (referred to as “surprise”). We calculated all bursts in the entire spike train and then applied this method to determine the onset of the burst, we then calculated the mean discharge rate determined by number of spikes from the period which fixation on the initial position started (20–150 ms) which is a period which the start of visual burst definitely falls within. Once we determined the burst onset time for every trial, we then obtained the average it to obtain the burst onset time estimate and the standard deviation.

We previously used a sliding window analysis to look at the transition of spatial codes in individual time epochs in reactive saccades (Sadeh et al., 2018) and in another paper on SC memory delay currently in preparation, and in both cases there was a transition from visual to motor codes. We do not do this here to compare the tasks because the timing of the tasks is so different it would be difficult to normalize them to the same time scale. So instead we focused here on fixed visual and motor windows.

### Spatial Analysis of Neuronal Response Fields: The TG Continuum

Visual and motor RFs were obtained for each neuron (using the temporal windows described above) and analyzed using a method that has previously been described several times (Keith et al., 2009; DeSouza et al., 2011; Sadeh et al., 2015; Sajad et al., 2015). Briefly, the RF of the neuron was plotted by overlapping firing rate data over two-dimensional position data corresponding to the spatial parameter related to the given model, such as final gaze position relative to the eye. The quality of the model for the data was quantified by calculating the Predicted Sum of Squares (PRESS) residuals for all trials, which is a type of cross validation in regression analysis (Keith et al., 2009). Specifically, the PRESS residual for a single trial was obtained by: (1) eliminating that trial from RF data; (2) fitting the remaining data points non-parametrically using Gaussian kernels at various bandwidths (2–15°); and (3) obtaining the residual between the fit and the missing data point. The overall predictability power of the model for the recorded data set was quantified by the average of PRESS residuals across all trials for that neuron. Once PRESS residuals of all the spatial models were obtained the spatial code of a neuron was then defined as the model (at the kernel bandwidth) that yielded the overall best fit (i.e., smallest residual) to the data. In order to characterize the spatial coding of the population of neurons the final step of our analysis involves combining the results of individual neurons in order to obtain the best fit model of that population (Keith et al., 2009).

Our previous studies have tested various spatial models of SC activity but have found the spatial continuum spanning the location of the target and the eventual gaze endpoint (i.e., *target-gaze (TG) continuum*) defined in eye-centered coordinates to be most useful in distinguishing visual from motor coding (Sadeh et al., submitted). The physical basis of the TG continuum is illustrated in Figure 1C, which shows the TG continuum for an example trial in space coordinates, which would look similar when rotated into eye coordinates (Klier et al., 2001). This continuum extends between, and beyond T and G position for every such trial, based on our behavioral measures. As described previously (Sadeh et al., 2015; Sajad et al., 2015), in our analysis the TG continuum was constructed by extending the possibility of the best fit for neural activity between and beyond target and gaze models within the same reference frames (eye coordinates). The intermediate spatial models were constructed by dividing the distance between target position and final gaze position for each trial into 10 equal intervals and 10 additional intervals extended on either end. Depending on the location of a neuron on the continuum a value (here referred to as TG alpha value), between 1 and 31 (the Target and Gaze locations are arbitrarily numbered 11 and 21, respectively) which indicates their relative preference for coding target vs. gaze related spatial information. For example, if the fit and TG continuum analysis for the activity of a given neuron yields the value of 11, this indicates that the spatial information encoded by this neuron’s activity is regarding the target location information rather than gaze endpoint information. Once the optimal TG value is determined, it can then be used to plot each neural RF in its intrinsic coordinate system, by plotting activity for each trial according to its location along the TG continuum (in eye-centered coordinates).

This TG continuum analysis is insensitive to systematic gaze errors and will automatically adjust to any magnitude of variable error, so long as the range of these errors sufficiently exceeds the noise range of the gaze recording system. In our previous papers we reported a variable error range of 0.7–10.8° for the Reactive Task and 1.3–12° for the Memory Delay task, both of whichreported a variable error range of exceed the level of noise in our recording system by more than an order of magnitude. The recording noise would thus show up as small constant residuals in the model fitting algorithm, and thus have little influence on the T-G comparison to comparison between tasks.

## Results

### General Observations

Of 86 neurons sampled on-line, we recorded complete datasets (in both the *reactive* and *memory delay task*) from 74 SC neurons from the left and right SC of two head unrestrained monkeys. Of these 54 neurons met all our inclusion criteria (Sajad et al., 2015), including 15 visual, 28 VM and 11 motor neurons (as identified using the memory delay task; Sadeh et al., 2015).

Figure 2 shows the activity profiles of each category of neurons in our study (Visual, VM, Motor) during gaze saccades to the top 10% data (corresponding to the RF “hot spot”) derived from the *reactive task (red) and memory delay task (black)*. Each panel provides mean spike density plots (averaged across neurons ± SEM). Data are aligned both with target onset (Left column; Figures 2A,C,E) and when aligned with gaze onset (Right Column, Figures 3B,D,F). By definition, visual neurons only showed a target-aligned response in the memory delay task (Black data in Figures 2A,B), whereas VM cells showed both visual and motor responses in both tasks (Figures 3C,D), and motor neurons only showed peak saccade-aligned responses (Figures 3E,F). Henceforth we will refer to the data from our fixed target and saccade-related windows as “visual activity” and “motor activity,” based on their temporal profiles, but later we will use our analysis methods to quantify what spatial parameters these activities encode in different neurons and at different times.

### Temporal Analysis of Reactive vs. Memory-Delay Population Activity Profiles

**Figure 7:**
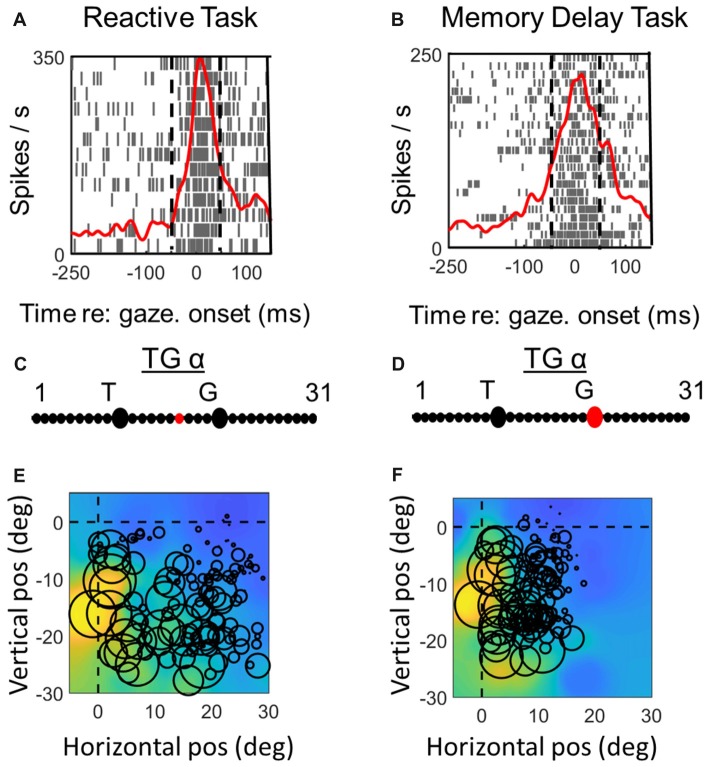
Spatial analysis of *motor* activity during reactive task (left column) vs. memory delay task for one example *motor* neuron. Vertical dashed lines in **(A,B)** represent the fixed motor analysis windows (−50 to + 50 ms relative to gaze onset). Otherwise the plotting conventions are the same as in Figure 3.

Although our main aim was to compare spatial tuning in neurons between the reactive and memory tasks, we also took the opportunity to compare their temporal firing profiles (Figure 2; selected individual examples are provided below in Figures 3, 4, 6, 7). In general, visual neuron responses (Figure 2A) and motor neuron responses (Figure 2F) showed similar response profiles in the *reactive* (red) and *memory delay* (black) tasks. However, there were some notable differences such as generally stronger peak activation in the reactive task, followed by a more robust, complex, and prolonged “tail.” To quantify the degree of response similarity across tasks, we performed a Pearson bivariate two tailed correlations through time on the population activity, and a paired two tailed *t*-test test to compare the peak top 10% of neural firing rate within the defined windows, in the visual and/or motor alignments as appropriate. Visual neurons (in the visual alignment) showed correlation of 0.934 between the two tasks for the fixed window analysis (*p* < 0.0001). But the peak activities were significantly higher in the reactive tasks (237 + 23 SD vs. 175 + 15 SD in *MD* task, *p* < 0.0001). Motor neurons (in the motor alignment) showed correlation of 0.90 (*p* < 0.0001; fixed window), but again had significantly higher firing rate in the reactive task (116 + 19 SD vs. 100 + 12 SD in *MD*, *p* < 0.0001). Thus, visual and motor profiles were highly correlated between the two tasks, but the peak responses were higher in the reactive task.

In contrast, VM cells (Figures 2C,D) showed very different profiles in our two tasks, presumably because the reactive burst contains both visual and motor activity. This is most evident in the visual alignment, where the *memory delay task* yields a burst that aligns well with the initial burst of activity from the *reactive task* data, but the latter shows an additional delayed peak that is presumably the motor response. Further, the visual response in the memory paradigm now seems higher, if anything. In the saccade alignment (D), the reactive task produced heightened earlier activation that could correspond to visual activation, but also a higher and more persistent motor peak that is harder to account for. When we repeated our statistical tests on these data (restricted within the fixed visual and motor temporal windows), we found lower, but still significant correlations in both the visual and motor windows (*r* = 0.7634 and 0.8164 respectively, with *p* < 0.0001 for both). There was no significant difference in the peaks of activity in the visual window of VM neurons (155 ± 10 in reactive, 150 ± 39 in *MD*, *p* = 0.1195), but a significant difference in peak activity in motor window (153 ± 15 in reactive vs. 103 ± 14 in the *MD* task, *p* < 0.0001). Thus, if we only look within the fixed visual or motor response windows of VM neurons, they again look somewhat similar and are highly correlated, but with differences in the gain of the motor (not visual) response.

Using the Poisson analysis approach, we found that the average onset of the visual burst in visual neurons in the reactive task is 65.24 ms (SD: 20.5 ms) relative to target onset and 64.6 ms in the MD task (SD: 21.9) with a range of 48.11–78.46 ms in reactive and 53.21–85.11 ms in MD task. The difference of burst onset was found to be non-significant (*p* = 0.8099, two tailed paired *t*-test). For the VM neurons the onset of visual burst was found to be 68.18 ms (SD: 20.5) relative to target onset in the reactive task and 67.78 ms (SD: 19.1) in the MD task, with a range of 54.71–84.79 in reactive and 57.12–88.36 ms in the MD task. The burst onset differences were also found to be non-significant (*p* = 0.6414, two tailed paired *t*-test). Furthermore, the onset of visual burst was not significantly different between the visual neurons and the VM neurons in reactive (*p* = 0.154, two tailed unpaired *t*-test) and MD (*p* = 0.2259, two tailed unpaired *t*-test) tasks.

Overall comparing the reactive task to the *MD* task, visual responses showed the same sharp rise but peaked higher in (in visual, not VM neurons), whereas motor responses were higher and more prolonged in both VM and motor neurons. In general, these results were consistent with similar temporal analyses of activity profiles that have been performed previously in head-restrained studies (Goldberg and Wurtz, 1972; Wurtz and Goldberg, 1972a; Sparks, 1978; Mays and Sparks, 1980; Munoz and Wurtz, 1995a,b; Dorris et al., 1997; Everling et al., 1999; Gandhi and Katnani, 2011), except that, as expected from past studies (Freedman and Sparks, 1997a; Crawford et al., 2003; Stuphorn, 2007; Walton et al., 2007; Freedman, 2008; Sadeh et al., 2012), our head-unrestrained responses were more prolonged and complex.

It is possible that head movements modulate the neural responses and possibly the spatial codes (Cowie and Robinson, 1994; Klier et al., 2002; Klier and Crawford, 2003; Corneil et al., 2007; Stuphorn, 2007; Walton et al., 2007). To ensure that the head movements are comparable for our spatial code comparison, we compared the head contribution to gaze (Head amplitude/Gaze amplitude) in MD (mean: 0.1011, SD: 0.1291) and reactive (0.1103 SD: 0.1301) and found no significant difference between the two (*p* = 0.61). We also compared the onset of head movements relative to onset of eye movements in reactive and MD tasks, where positive (+) indicates head onsets after the saccade onset. Head movements occurred after eye movements in both tasks, but movement onsets were not significantly later (+64 ms ± 33.3 ms SD) in the reactive task compared to the MD task (+52.5 ms ± 36.8 ms SD) (*p* = 0.09, unpaired *t*-test). This difference might influence the late temporal profiles described above (see “Discussion” section). However, our previous analysis did not find any neurons that showed a preferential code for head position or displacement during our visual or saccade-related analysis windows (Sadeh et al., 2015). The following *spatial* analysis exclusively focuses on those windows and only differentiated target vs. gaze coding.

### Comparison Between Spatial Coding in Reactive vs. Memory Delay Tasks: Visual Responses

The preceding temporal analysis suggests both similarities and differences in the Visual and Motor responses to our two tasks, but this itself does not indicate whether the same or different spatial information is being encoded. As noted in the introduction, it is likely that visual and motor responses interact in the *reactive task*, and that suppression and memory signals are present in the *MD* task (White et al., 1994; Brown et al., 2004). These factors could affect not only the vigor of the responses (described above) but also their spatial code. To test these various assumptions an independent criterion is required. Here, we did this by using the TG alpha continuum as an independent test of spatial coding in various points of these tasks. Note that for the TG-alpha analysis, all trials were used (not just those to the “hot spot,” so this analysis is based on a much larger, richer dataset.

Figures 3, 4 compares spike density and raster plots (A vs. B), best fits along the TG continuum (C vs. D) and visual receptive fields plotted in these ideal coordinate frames (E vs. F) tested on the same stimulus locations with the reactive task (left column) vs. the *MD* task (right column), for an example visual neuron (Figure 3) and the visual response of an example VM neuron (Figure 4). These representative visual neuron raster plot (Figure 3) resembles the population raster seen in Figure 2, with a more prolonged burst and a more prominent second burst in the reactive task. However, the best TG fit for the visual response is shifted leftward toward T in the memory delay task. The overall location and shape of RF remains similar in the two tasks, despite some slight distortions in stimulus location caused by the change in coordinate frame used for the plot. The Visual response of the example VM neuron (Figure 4) showed similar patterns, except that the relative TG shift was in the opposite direction.

To assess whether these TG shifts followed a pattern (or were randomly distributed across neurons) we compared fits for the two tasks across our entire neuron populations. Figure 5 does this for the visual neurons population (left column), and visual response of the VM population (right column), providing frequency histograms for TG-alpha fits from the *reactive task* (top row; Figure 5A,B) and *MD task* (middle row; Figure 5C,D), recorded from the same neurons (recall that TG = 11 denotes a pure target code, whereas 21 denotes a pure gaze code). For this analysis, we used the fixed target-aligned window to compare the two tasks. The mean TG alpha value for visual neurons in the reactive task was 12.2 (SD = 4.35) which was not significantly different from 11.87 (SD = 2.42) in the memory delay task (*p* = 0.91, paired *t*-test). This indicates a slight, but not statistically significant, shift toward coding target location in the *MD* task. In the visual activity of VM neurons the same trend (mean reactive: 12.4 SD = 3.4, *MD*: 10.9 SD = 4.6) is observed with a borderline non-significant (*p* = 0.058, paired *t*-test) shift toward target coding in the *MD* task.

To directly visualize these comparisons we plotted the TG alpha values of each neuron in the *MD* task (x axis) and the reactive task (y axis), For the visual neurons (Figure 5E) there is an almost equal number of neurons which do (*n* = 7) and do not (*n* = 8) have different spatial coding between the tasks, in visual activity of VM neurons (Figure 6F) more neurons (*n* = 21) do not have a change of what spatial info is being encoded in MG vs. the reactive task. When we combined the TG values all the visual activity (i.e., visual neurons and the visual activity of VM neurons) and compared them between the two tasks there is a slight, but not significant (*p* = 0.34, paired *t*-test), preference for target coding in *MD* (mean TG = 11.53 ± 4.45) compare to reactive task (mean 12.33 ± 3.73). Finally, there was no significant difference between the goodness of fit (i.e., the mean PRESS residual) of these models to the visual data across all visual and VM neurons in the reactive vs. *MD* tasks (*p* = 0.89, paired *t*-test). In summary, there was no significant task-dependent difference in spatial coding for the visual responses of the visual and VM neuron populations.

### Comparison of Spatial Coding in Reactive vs. Memory Delay Tasks: Motor Responses

Figures 6, 7 (similar to 3 and 4) provide comparisons between the spike density plots, rasters, and non-parametric best fits of motor response fields tested on the same stimulus locations, for the motor response of an example VM neuron (Figure 6) and motor neuron (Figure 7), respectively. The motor burst of the VM neuron (Figure 6, top row) illustrates trends seen in the population (Figure 2), being completely separated temporally from the visual burst in the *MD* task (Figure 6B) but not the reactive task (Figure 6A). For this neuron, the TG fits for the motor response are quite close to G in both tasks (Figures 6C,D). As a result, the motor RFs for this neuron (which shows a fairly poor spatial organization) looks nearly identical in both tasks (Figures 6E,F).

In the case of the motor neuron example, the TG fit is shifted more toward G in the *MD* task (Figures 7C,D). As a result, the saccade end locations for the RF plot are slightly compressed in the horizontal dimension for the *MD* data, i.e., because of gaze undershoots in this task. But otherwise, the RFs are similar, showing a downward-leftward preference.

**Figure 8:**
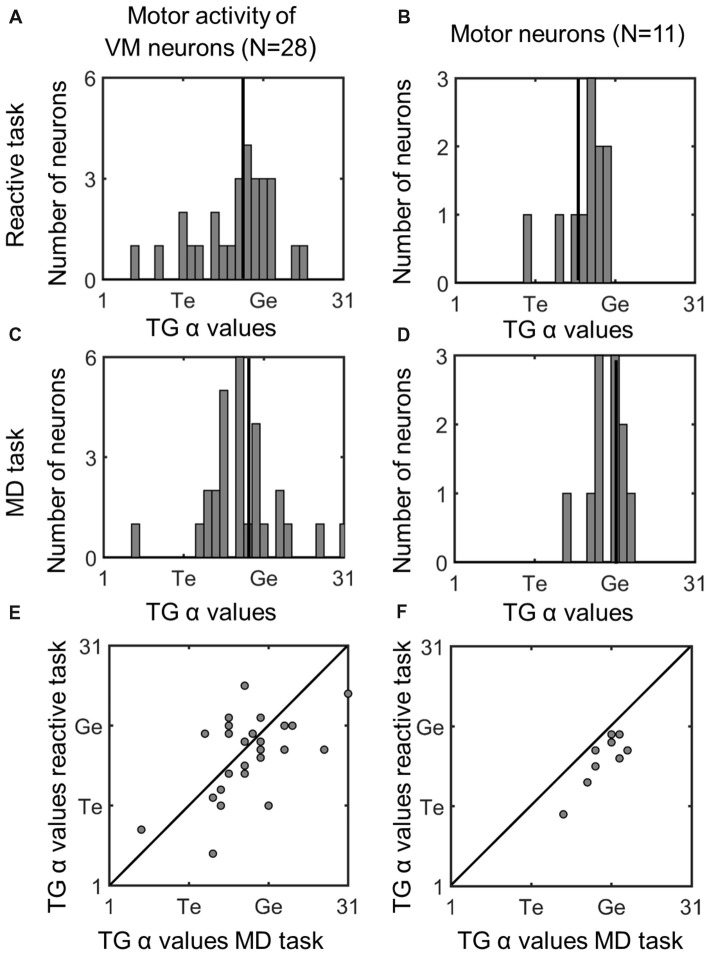
Comparison of TG continuum coding of reactive vs. memory delay task in *motor* responses. *Left column* **(A,C,E)**: motor response of VM neurons; *right column* **(B,D,F)**: motor response of motor neurons. Otherwise, the conventions are the same as Figure 5. There was a significant difference between the TG values in the two tasks in Motor Neurons, i.e., the fits were below the diagonal line in part (F) meaning motor neurons were more gaze-related in the MD task.

Figure 8 (similar to Figure 5) compares the TG alpha values for the fixed window motor responses of VM and Motor Neurons. The motor activity of the VM neurons showed more gaze preference in the *MD* task (mean: 18.3; SD = 4.8) than reactive task (mean = 17.6; SD = 4.9), but this was not significantly different (*p* = 0.58). Interestingly in our pure motor neurons the TG alpha values showed a significantly (*p* = 0.021, paired *t*-test) more preference (mean: 20; SD = 2.29) for coding the gaze end points in the MG task compare to the reactive task (Mean: 17.2; SD = 2.9). This can be visualized in Figure 8F as a shift in the data from the line of unity, whereas the VM data (Figure 5E) are evenly distributed across this line. The TG values for the combined motor activity, however, were not significantly different between the two tasks (*MD* task mean = 19 ± 4.2, reactive task mean = 17.5 ± 4, *p* = 0.08). In the case of these motor fits, there was a significant increase in the mean PRESS residual across cells (*p* = 0.03), for the *MD* vs. reactive task, possibly because the delay introduced more non-spatial noise in the system. Thus, overall our data tend to confirm the assumption that what is spatial encoded in motor responses of VM neurons is the same regardless of the interposition of a memory delay, but suggests that motor cells show a purer gaze code following a memory delay.

## Discussion

Gaze shifts occur in a variety of circumstances (e.g., exploring the environment, selecting the stimulus of interest, looking at a suddenly appearing stimulus, etc.) and in each case the number of brain VM areas and the extent of their involvement in generating gaze shifts are different (Fischer, 1986; Dean et al., 1989; Gnadt et al., 1991; Pierrot-Deseilligny et al., 1991a,b; Andersen, 1995; Deubel, 1995; Horwitz and Newsome, 1999; Hikosaka et al., 2000; Schall, 2001; Brown et al., 2004; Fecteau and Munoz, 2006; Sajad et al., 2016a). It is, however, unclear if these differences in the task demands and behavior has any influence in the spatial information encoded by the neurons.

Here, we compared the visual and gaze movement related neural activity during head-unrestrained reactive and memory delay gaze tasks in the SC, a key oculomotor area where many signals from cortex and subcortical areas converge and which directly influences the brainstem premotor neurons that control eye and head rotation (Guitton et al., 1980; Harris, 1980; Sparks and Hartwich-Young, 1989; Klier et al., 2002; Sparks, 2002; Klier and Crawford, 2003; Walton et al., 2007; Gandhi and Katnani, 2011). We found both similarities and differences between the overall activity profiles and spatial information encoded by SC neurons between the memory guided and the reactive gaze shifts.

### Differences in the Timing and Vigor of Visual and Movement Related Neural Responses

By comparing spike density profiles recorded from the same neurons in two different tasks (Figure 2), we were able to make several noteworthy observations. As expected, the onset and peaks of the visual responses occurred at roughly the same time after target presentation, and the peak of the motor response was similar in both tasks. There was also a strong correlation between the peaks in each task. However, visual neurons showed a higher peak firing rate and all motor responses were higher, as reported in several previous studies (Goldberg and Wurtz, 1972; Mays and Sparks, 1980; Krauzlis et al., 2013). This might be accounted for by the presence of saccade suppression signals during the early portion of the *MD* task, and conversely, the presence of visual target and increased bottom-up attention in the reactive task (Desimone and Duncan, 1995; Itti, 2005; Buschman and Miller, 2007).

Effects of offset timing were pronounced in the head-unrestrained condition, where the visual and motor bursts are already prolonged to accompany the longer duration of the eye + head motion (Freedman and Sparks, 1997a; Roy and Cullen, 1998; Freedman, 2008). For example, the motor burst, already prolonged in head-unrestrained gaze shifts, was even more prolonged in the reactive task than the *MD* task. This and the prolonged, multi-peaked burst activity of visual neurons in reactive task could be attributed to modulations such as attention (Goldberg and Wurtz, 1972; Desimone and Duncan, 1995; Robinson and Kertzman, 1995; Krauzlis et al., 2013), motivation (Redgrave et al., 2010; Otmakhova et al., 2013). The reactive task trials were shorter so monkeys were rewarded at a higher rate compared to the *MD* task, suggesting that reward signals may have also had an influence (Glimcher and Sparks, 1992; Schall, 2001; Ikeda and Hikosaka, 2003). These differences in firing could account for differences in speed (Tweed and Vilis, 1990; Lefèvre et al., 1998; Groh, 2001; Sparks, 2002), accuracy (Lee et al., 1988; Goldberg and Bruce, 1990; Gottlieb and Goldberg, 1999) and amplitude (Sparks et al., 1990; Dorris et al., 1997; Freedman and Sparks, 1997a,b) reported here and previously in these tasks. The differences in gaze precision reported here (see “Materials and Methods” section) might also be due to lower signal-to-noise ratio in the *MD* firing rates, related differences in spatial coding, which we will describe more directly in the following sections.

Finally, task-related head movement strategies could modulate SC responses (Guitton et al., 1990; Cowie and Robinson, 1994; Crawford and Guitton, 1997; Crawford et al., 1999, 2003; Sparks et al., 2001; Corneil et al., 2002; Klier et al., 2002; Corneil et al., 2007; Walton et al., 2007; Freedman, 2008). There was no significant difference in head contribution to the gaze shift between our tasks, but head onset occurred earlier in the MD task. This is in agreement with prior observations of eye head coordination timing during various tasks and conditions (Berthoz and Grantyn, 1986; Guitton, 1992; Freedman and Sparks, 1997b; Crawford et al., 1999; Pélisson et al., 2001; Corneil et al., 2002; Snyder et al., 2002). It is possible that head related activities influence the neural response differently in each activity (Cowie and Robinson, 1994; Sparks et al., 2001; Klier et al., 2002; Klier and Crawford, 2003; Knight and Fuchs, 2007; Stuphorn, 2007; Walton et al., 2007; Freedman, 2008; Monteon et al., 2012). Specifically, this timing difference might explain the more prolonged motor burst we observed in the reactive task. However, it is unlikely to explain the higher peak in the saccade-related response. Further, it is unlikely to have influenced the spatial analysis described in the next sections because; (1) this analysis focused on target vs. gaze coding during the earlier visual and saccade epochs; (2) head movements started from a central range and tended to be quite small (Sadeh et al., 2015); and (3) we found no neurons that preferentially encoded head movement or position during these epochs (Sadeh et al., 2015). It remains possible that some of these neurons might predict or code head movement in tasks that involve larger head movements and/or offsets (Walton et al., 2005, 2007; Monteon et al., 2012).

### Spatial Code Differences in Visual Activity

In both the reactive and *MD* tasks the visual activity preferentially coded for Te, and there was no significant difference between these codes. However, there were subtler differences in the distribution of the TG alpha values of the visual responses between the two tasks (Figures 5A,C). In visual neurons, the distribution of the TG values was clearly more narrow and clustered around the Te model in the *MD* task compared to the reactive task (Figures 5A,C). In VM neurons, the peak of the TG distribution was shifted slightly (although not significantly) toward Te. Overall, this suggests a more faithful coding of the target in visual responses in the *MD* task.

This could be due to an almost simultaneous need for encoding target location as well as preparing for the movement in the reactive task, thus some of the visual responses may have been influenced by movement preparation (Munoz and Wurtz, 1993; Dorris et al., 1997; Horwitz and Newsome, 1999; Bell et al., 2005) which may shift the spatial information away from the Te model. In the *MD* paradigm when a delay is expected, the visual burst encodes the target location and the signals regarding the movement preparation and from the working memory circuit contribute to the later movement related burst and therefore less intermixing of the activities occurs. This could occur between suprathreshold excitatory motor signals in VM neurons, or through subthreshold or inhibitory motor signals in visual neurons.

### Spatial Code Differences in Motor Activity

In both tasks, the motor related response of the VM neurons tend to encode spatial information related to gaze endpoint locations rather than target, but this shift toward G coding was more complete in pure motor cells, with closer clustering around the G model in both tasks (Figures 5B,D). This is consistent with our previous results from the frontal eye fields, where moto-only cells showed a pure G code (Sadeh et al., 2015). Since our method of fitting G is based on fitting variable errors in gaze end points, this suggests that SC motor responses, particularly in pure motor cells, are casually involved in generating these errors in both tasks, perhaps in communication with other areas like the FEF (Sajad et al., 2015). Alternatively, pure motor cells may receive feedback from downstream premotor cells that provide a better estimate of actual behavioral output (Waitzman et al., 1991; Matsuo et al., 2004; Walton et al., 2005).

In addition, there was a significantly further shift toward G coding (in pure motor cells) in the *MD* task (Figures 5B,D,F). Our model normalizes fits relative to the magnitude of errors (Keith et al., 2009; Sajad et al., 2015; Sadeh et al., 2015), but the quality of the fits could have been influenced by signal-to-noise ratio. However, this does not account for why this task-dependance occurred only in motor neurons and not VM neurons. One possible explanation is that by the time motor neurons became active after the memory delay, there was less influence from other neurons with mixed coding on behavior (perhaps through selective gating), and thus an even better relationship between their firing rate and gaze errors. Alternatively, if we consider feedback, it may be that more delay allows a more accurate estimate of output. Other more general explanations will be considered in the “Spatial Transformation in Reactive and Memory Delay Tasks” section.

### Spatial Transformation in Reactive and Memory Delay Tasks

In both the reactive and *MD* tasks the general observation is that based on the significantly different TG values, there is a transformation away from coding the location of target to coding the location of gaze end points. In our previous articles, we have suggested that this is due to the accumulation of noise in the system (Sadeh et al., 2015, 2018) and as confirmed here, this appears to happen with or without the interposition of a memory delay.

However, despite this similar trend there are some interesting findings which suggest different VM transformation mechanism in each task (Gnadt et al., 1991; Munoz and Everling, 2004; Sajad et al., 2016a,b). As noted above, the visual code represents the target more faithfully in the *MD* task (in terms of overall distribution), whereas the motor response (at least in pure motor cells) more faithfully represents the gaze end point. This would seem to suggest a more perfect transformation, and yet gaze saccades are less accurate and precise after a memory delay in our data and in previous studies (Gnadt et al., 1991; Pierrot-Deseilligny et al., 1991a; Stanford and Sparks, 1994; White et al., 1994).

The answer to this apparent contradiction may be that, despite the rapid mixing of visual and motor signals in the reactive task, at the overall population level the transformation is relatively effective. Second, although the final motor output in the *MD* task faithfully encodes gaze, this includes gaze errors, and the SC (and FEF) is likely one source of these errors, or as suggested above, in the monitoring of those errors as movement progresses.

Finally, the transformation is not necessarily complete at the SC. For example, the TG values of motor activities are significantly different from that for the gaze model, shifted towards the target model, and in the *MD* task they are not. Therefore, it is reasonable to conclude that the SC encodes a gaze goal, which is then subject to further transformations. Errors in those additional transformations could be proportionately larger in the reactive task than the *MD* task, hence the difference in motor code between these tasks. Alternatively, this difference could be attributed to the increase in distribution of errors with time across the entire gaze control system, hence any one area like SC could reflect the gaze errors more closely.

Finally, the current study demonstrates that the model-fitting approach used here is sufficiently robust to identify not only differences in neural codes and transformations (Sajad et al., 2015, 2016a; Sadeh et al., 2015, 2018), but also how these depend on brain states. The memory delay interval is known to induce inaccuracies in motor response in a variety of the settings (Postle et al., 2000; Bays et al., 2011; Barber et al., 2013; Chatham and Badre, 2015; Hollingworth, 2015), but so do other behaviors like express saccades (Pierrot-Deseilligny et al., 1991b; Postle et al., 2000; Corneil et al., 2004). More importantly, one would expect such errors to be even larger in clinical disorders (Munoz et al., 2003; Anderson and MacAskill, 2013), so this technology might have practical application for detecting quantitative biomarkers in disease states.

## Conclusion

In this article, we aimed to provide a comprehensive comparison between the spatial information encoded by visual and motor activities of SC in two different tasks: reactive and memory delay gaze shifts. We found that despite overall similarities in visual to motor transformation, there are several important differences. Most importantly the visual to motor transformation is more extensive in the *MD* task since the TG values in the motor population are closer to gaze models in the *MD* task. This suggests that the brain areas involved in each task contribute to changes in spatial code and ultimately to saccadic errors.

## Author Contributions

MS performed experiments, assisted in surgeries, analyzed data and wrote the article. AS assisted in data analysis and edited the article. HW performed surgeries, assisted in experiments and edited the article. XY set-up the equipment, assisted in experiments and edited the article. JC designed and funded the experiment, provided advice on experiments and analysis and co-wrote the article.

## Conflict of Interest Statement

The authors declare that the research was conducted in the absence of any commercial or financial relationships that could be construed as a potential conflict of interest.
